# Increasing landscape heterogeneity as a win–win solution to manage trade-offs in biological control of crop and woodland pests

**DOI:** 10.1038/s41598-023-40473-2

**Published:** 2023-08-21

**Authors:** Axelle Tortosa, Brice Giffard, Clélia Sirami, Laurent Larrieu, Sylvie Ladet, Aude Vialatte

**Affiliations:** 1 Université de Toulouse, INRAE, DYNAFOR, Castanet-Tolosan, France; 2 Bordeaux Sciences Agro, INRAE, ISVV, SAVE, 33140 Villenave d’Ornon, France; 3 CNPF-CRPF Occitanie, 7 chemin de la Lacade, 31320 Auzeville Tolosane, France

**Keywords:** Ecosystem services, Ecosystem ecology

## Abstract

Agriculture and forestry cover more than 75% of Europe, and invertebrate pests are a costly challenge for these two economic sectors. Landscape management is increasingly promoted as a solution to enhance biological pest control, but little is known on its effects on adjacent crop fields and woodlands. This study aims to explore the effect of the proportion of woodlands and permanent grasslands as well as crop diversity on biological pest control simultaneously in cereals fields and woodland patches, in south-western France. We used different types of sentinel prey as well as bird and carabid community metrics to assess biological pest control potential in these two ecosystems. We first show that land cover variables influence biological pest control both in cereal fields and woodland patches, but have antagonistic effects in the two ecosystems. Although results vary according to the biological control indicator considered, we show that increasing landscape heterogeneity represents a valuable solution to manage trade-offs and promote higher average predation rates across forests and cereal fields. Our study therefore calls for more integrative studies to identify landscape management strategies that enable nature-based solutions across ecosystems.

## Introduction

Arthropod pests are a costly challenge for both farmers and foresters. Indeed, arthropod pests are responsible for a 20% yield decrease on average across crop types^1^ and are a major threat to forest health, with severe outbreaks that are likely to worsen due to climate change^2^. Pest control solutions based on technology such as pesticide use or genetic selection are increasingly showing their limits^3,4^. An alternative but non-exclusive way is to consider nature-based solutions, in particular biological pest control^5–8^. However, agricultural intensification, deforestation and landscape simplification over the last decades have resulted in a dramatic loss of biodiversity in agricultural landscapes, both in agricultural fields and semi-natural ecosystems such as forests, meadows or rivers^9–12^. Such biodiversity loss hampers the ability to rely on nature-based solutions. It is therefore crucial to assess to which extent managing landscapes may enable nature-based solutions such as biological pest control across ecosystems.

The effect of land cover variables on biological pest control (as phytophagous insects and weeds) in crop fields has been extensively studied. Diversified crop mosaics support higher biodiversity and favour biological pest control in crop fields^13–17^. This effect is consistent with the hypotheses of resource complementarity for natural enemies^18^ and host concentration for pest species^19,20^. The benefits of woodland and grassland covers, and more generally semi-natural habitats (SNH—e.g. hedgerows, shrublands, riparian edges), are also consistent with the fact that they provide complementary food resources, shelters, nesting and overwintering sites, for natural enemies^21–23^, which result in higher biological pest control within crops^14,23^. However, the effect of SNH cover may vary across agronomic settings and taxa^24–26^. For instance, forest cover appears to favour both some rapeseed pests and their natural enemies^27^. Furthermore, field-level farming practices and weed diversity are known to influence biological control, and may also modulate the effects of land cover variables on biological control^28–30^.

Much less is known about the effect of land cover variables on biological control within forest patches in rural space. Like within crops, local forest management and tree diversity are well-known to influence the level of biological control of forest pests^31,32^ mainly due to defoliator caterpillars^33^. However, unlike within crops, little is known about the effects of adjacent non-forest ecosystems on biological control within forest stands. This is primarily due to the dominance of a binary representation with forest patches surrounded by a hostile agricultural matrix (e.g.^34^). However, this patch-matrix paradigm is now considered outdated^35,36^. Indeed, land cover types surrounding forest patches are likely to influence biodiversity occurring within these patches^37^, including forest pest’s natural enemies such as birds^13,38^. For instance, crop fields and grasslands are likely to provide complementary resources for bird predators occurring in small forest stands^39–41^, and to influence the spillover of arthropod predators from crops and grasslands to forests^39,42^.

Promoting biological control within and even more so across different ecosystems is likely to be challenging, because it is highly context-dependent, involving a complex combination of factors at local and landscape scales^43,44^. Synergic and antagonistic effects may occur between these factors. For instance, local pesticide use intensity within fields may hamper positive effects of SNH on biological pest control^30^, whereas plant diversity promotes natural enemies and biological control at the field and landscape level^45,46^. Towards forest patches, many studies showed that biological control in forests is mainly related to bottom-up effects through tree diversity and neighbour tree density^32,47–49^. It has also been shown that forest cover within landscape increases biological pest control^13^, but no study has yet explicitly looked at the combined effects of these factors. Thus, in order to evaluate the potential synergic or antagonistic effects between ecosystems, it seems necessary to move to a higher level through a landscape approach integrating these different ecosystems. Moreover, many studies demonstrate that increasing landscape heterogeneity positively influence pest predation^50–52^. We hypothesized that considering landscape heterogeneity would allow to manage potential antagonistic effects of land cover variables combined with specific local context between ecosystems and to bring out trade-offs.

In this paper, we explore the effect of land cover variables on biological pest control in both woodland patches and cereal fields in south-western France. In a first step, we use an ecosystem-level approach in order to test the hypothesis of resource concentration, i.e. the effect of the proportion of land covered by the target ecosystem on biological control^19,20^, and resource complementarity, i.e. the effect of different land cover types on biological control^18^. We predicted higher amount of SNH cover such as woodland and grassland covers should promote higher predator diversity and potential predation. In a second step, we use a landscape-level approach to test the effect of land cover on the average biological pest control across both ecosystems.

## Material and methods

### Study site

The study was conducted in “*Vallées et Coteaux de Gascogne”*, a 370 km^2^ hilly area located in south-western France (43°17′N, 0°54′E). The study area, which is part of the Long-Term Socio-Ecological Research site LTSER ZA PYGAR, is dominated by mixed crop-livestock farming systems, and where grasslands and crop fields are interspersed by woodlands. Winter crops (mainly wheat, barley, rapeseed) are sown in autumn and harvested in early summer, and spring crops (corn, sorghum, sunflower) are sown in spring (without winter cover) and harvested at the end of the summer. Wheat is the dominant crop type and is traditionally grown in this region in a wheat–barley–alfalfa or a wheat–wheat–sunflower rotation. Woodlands in this study site are characterised by coppices with standards, oak-dominated deciduous stand and composed mainly of indigenous species^53^, and grasslands are mostly permanent cattle pastures with no fertilizers use, mown once a year and also with high levels of vegetation diversity^54^.

### Sampling site and landscape selection

We obtained the land-cover map of the study area by combining GIS layers from the Soil Occupancy Product OSO 2016^55^ and the Land Parcel Information System linked to the Common Agricultural Policy^56^ using ArcGis Desktop 10.5.1 software (ESRI, Redlands, CA, US)^57^. We identified annual crop types based on direct field observations in 2016 and the LPIS 2016. Crops were categorized in four categories: winter cereal crops, other winter crops, spring crops and perennial crops.

We selected 30 woodland patches along a gradient of woodland cover (2 to 40%) and winter cereal fields cover (0 to 41%). We also controlled for woodland size and age, two factors that have an impact on ecological processes and biodiversity^58,59^. The proportion of permanent grasslands ranged between 9 to 54% (Table S2). The size of woodland patches ranged from 1 to 5 ha. We made sure that all selected woodland patches were ancient woodlands, i.e. more than 50% of their area was characterized by a continuous presence of wooded vegetation. We used as reference the Etat-Major raster map established in 1850^60^, since it matches with the minimum forest cover in France. Thus, current forests already indicated on this map are considered likely to have never been cleared or replaced by another land use^61^.

We also selected 30 winter cereal fields along a gradient of winter cereal fields cover (13 to 67%) and woodland cover (0 to 39%). All fields were managed conventionally. The proportion of permanent grasslands ranged between 0 to 48% (Table S2). The mean size of fields around selected cereal fields was 4.4 ha (min: 0.39 ha; max: 18 ha). When possible, selected crop fields were located close to selected woodlands.

All the land cover metrics were calculated within a 500 m radius buffer as it is commonly used to assess biological pest control^3,50,52,62^ and/or natural enemies communities^29,63,64^.

**Figure 1:**
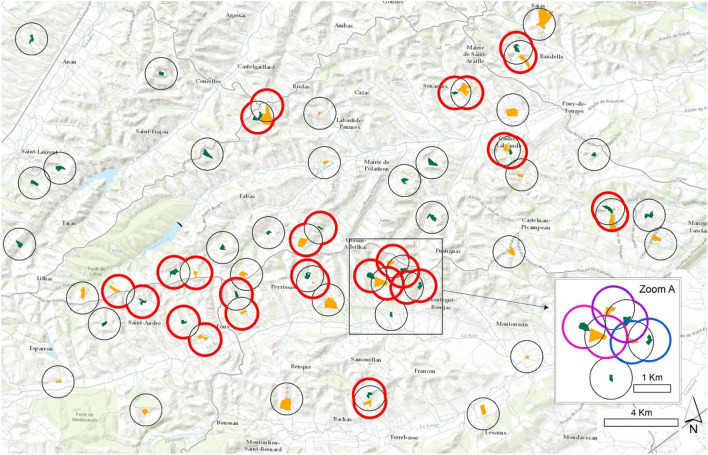
Location of the 30 woodland patches in dark green and the 30 cereal fields in yellow. The buffers circled in red correspond to the 15 matched landscapes grouping one cereal field and one woodland. The matched landscape buffer is the merging of the woodlands and cereal fields centred landscapes respectively. Zoom A shows a more detailed map of 3 overlapping landscapes using three distinct colours. The map was generated using ArcGis Desktop 10.5.1 software (ESRI, 2023—https://www.esri.com).

Finally, we selected 15 woodlands and cereal fields that were close enough to be considered as paired study sites located within the same landscape context (Fig. 1). We therefore call ‘landscape’ the combination of the two buffers centred on the cereal and woodland ecosystems respectively. When different combinations of woodland patches and cereals fields were available, we selected the combination with the most similar landscape context.

### Land cover variables

We explored the role of three land cover types (woodland, permanent grassland and crops) on biological pest control potential within each ecosystem. To do so, we used the proportion of woodlands, the proportion of permanent grasslands and crop diversity, three variables frequently used in landscape ecology^5,62,65,66^. We measured these three variables within a 500 m radius buffer around the centre of the 30 cereal fields and the 30 woodland patches. To measure crop diversity, we calculated the Shannon index of all crops, using the four crop categories listed above.

We then tested the effect of these land cover variables at the landscape level. To do so, we recalculated the three land cover variables for the 15 landscapes, within the area obtained after merging 500 m radius buffers around the woodland and the cereal field. As a result, this area varied between 1.3 and 1.9 km^2^ depending of the distance between the two paired study sites. In addition, we assessed the landscape heterogeneity by calculating the Shannon index on all land cover types defined above, which are woodland, permanent grassland and crops (i.e. winter cereal crops, other winter crops, spring crops and perennial crops). Note that we did not include landscape heterogeneity in our analyses at the ecosystem level because this variable was strongly correlated with crop diversity at that level. Details on land cover variables are presented in Supplementary material (Appendix D, Fig. S3 and Table S2).

### Local management and plant diversity covariates

We assessed two local indicators that are known to influence predators and pest biological predation in crop fields or woodlands and may influence the effect of land cover variables on pest biological predation: management intensity and plant diversity^45,48,67,68^.

In woodlands, assessing management intensity was challenging due to the multitude of woodland owners in the study area and the need to consider woodland management over a long period. We therefore used an indirect method based on the observed characteristics of woodland stands^69,70^, using four variables (see Supplementary Material) that are good proxies of woodland management^71,72^: lying deadwood, standing deadwood, very large live trees and microhabitats bearing trees (Table S1) and combined them using the following formula (1):

1 $$\mathrm{Management \; intensity}=\frac{1}{l \; deadwood+s \; deadwood+vl  \; live  \;trees+mb \; trees}$$

To assess plant diversity, we focused on the tree diversity. Within each woodland, we selected 3 to 5 plots of 26 m radius depending on the woodland size (Fig. S1). We identified all trees over 10 cm in diameter at breast height; we assessed the abundance of each tree species and we calculated the Shannon diversity of trees within woodland patches. All measurements took place in 2016.

In cereal fields, we interviewed farmers during the winter 2016–2017 to collect data on farming practices applied in sampled crop fields since sowing, i.e. between October 2015 and spring 2016. We then calculated the treatment frequency index (TFI) as a proxy of management intensity^73,74^. TFI combines data on all pesticide uses (insecticides, fungicides and herbicides) according to the following formula (2):

2 $${TFI}_{total}= \sum_{i=1}^{n}\frac{{D}_{i}\times {S}_{i}}{{Dr}_{i}\times S}$$

where ${D}_{i}$ is the applied dose, ${S}_{i}$ the treated surface area, ${Dr}_{i}$ the reference dose obtained from the French Ministry of Agriculture online database^75^ and $S$ the total area of the field for each spraying operation $i$^30^. Data required to calculate TFI were missing for five sampled cereal fields. We therefore used an imputation method to estimate TFI in these fields. We performed several imputation methods, including random imputation, the Multiple Imputation by Chained Equations^76^ and mean imputation. As results were similar across methods, we chose to keep the simplest method, i.e. to impute the average TFI of all cereal fields to these 5 sites. To assess plant diversity, we focused on the diversity of weeds and we placed 10 quadrats of 50 cm × 50 cm, positioned 50 m inside the field and spaced 5 m apart. We assessed plant cover and diversity in each quadrat and calculated the average Shannon index of weed community at the field level. All measurements took place in 2016.

We combined the two datasets on management intensity collected at ecosystem level to compute the average management intensity at landscape level. To do so, we averaged standardized variables in woodlands and crop fields: the management intensity in woodlands and the management intensity in cereal fields.

### Bird and carabid surveys

We selected two components of biodiversity that are known to play a key role in biological pest control in crop fields and/or woodlands: birds and carabids. Indeed, numerous bird species are potentially effective biological control agents in both crop fields and woodlands^13,40^, while carabids are considered as key agents in biological pest control in crop fields^77,78^. We therefore consider these two taxonomic groups as proxies of biological control.

In woodlands, we conducted 15 min points-counts, during which we recorded all birds heard within a distance of 100 m to make sure that all birds recorded were located within woodland patches (Fig. S1). We conducted 2-point counts: in late April 2016 when early nesters are active, and in late May 2016 when migratory species are present. We kept the highest abundance of each species between the two sampling periods and calculated for each woodland patch, the total bird abundance and the Shannon diversity index for the whole community as well as the Shannon diversity index for strict insectivorous species.

In cereal fields, we conducted 15 min points-counts, during which we recorded all birds only heard within a distance of 200 m in crop fields (Fig. S2) because open habitats hosts larger and more mobile species^79^. Since our study aimed to compare the effect of landscape variables on bird communities in the two distinct ecosystems, using different buffer radii in the two ecosystems was not a problem, and it was more important to adapt our protocol and make sure we recorded the whole bird community likely to contribute to biological control in cereal fields. The mean size of crop fields is smaller than the 200 m buffer but birds detected outside of the crop fields are likely to come into the fields. Bird surveys conducted in cereal fields were therefore representative of the bird community associated with the crop mosaic (including small woodland patches hedgerows and other field margins in the vicinity of the field) rather than the wheat crop field itself. We conducted point counts once in late May 2016, as the majority of bird species is present at this date in open habitats of this study area^80^. Swallows (*Delichon urbicum, Hirundo rustica*) and pheasants (*Phasianus colchicus*) were excluded from further analyses as they are mostly associated with human infrastructures or are released for hunting in the study area. One cereal field was considered as an outlier since only one individual was recorded in the associated point count and this point was removed from further analysis on bird community metrics. We calculated for each crop field, the total bird abundance and the Shannon diversity index for the whole community as well as the Shannon diversity index for strict insectivorous species. In cereal fields, we also sampled carabid communities (Fig. S2). To do so, we used 4 pitfall traps, 2 of which were located 50 m away from the field border and 2 others 100 m away, during 2 sampling periods, at the end of April 2016 and the end of May 2016. We calculated the total abundance of each carabid species over the two sampling periods. We then calculated the total abundance and Shannon diversity of carabid beetles.

At the landscape level, we combined the two bird datasets collected at ecosystem levels: we used the highest abundance across woodland patches and crop fields for each species in order to avoid double counting in cases where the point-count buffer around the crop field overlapped with the sampled woodland. We then calculated the total abundance of birds at the landscape level, as well as the Shannon diversity index of all bird species and the Shannon diversity index of strict insectivorous species.

### Experiments on predation rates

We selected standard experiment protocols that are adapted to assess predation rate in woodlands and cereal fields^81,82^. Moreover, we implement these protocols in order to fit the specific context of our study area, in particular the type of pest that is most common.

In woodlands, we assessed predation using plasticine models mimicking lepidopteran pest larvae^82^. Each larva was made of green, inodorous plasticine and shaped to reasonably mimic caterpillars, e.g. *Tortrix viridana,* a species common in the south-west of France, present relatively early in the season and most regularly found in oak forests. Plasticine caterpillars were attached to a thin metal wire around branches of 3 shrubs/trees within each one of the three plots used to assess woodland management intensity. On each shrub/tree, we placed 3 plasticine caterpillars at different heights during 7 days at the end of May 2016 (Fig. S1). We then estimated avian predation rate as the proportion of caterpillars with bird predation marks within each woodland^83^.

In cereal fields, we measured potential predation using sentinel prey cards, a standardized method which allows detecting variations in the level of biological control^84,85^. We used different prey species (aphids, moth eggs and weed seeds) in order to monitor different types of predation, at the ground and crop level (see further details in Supplementary Material). Within each crop field, we selected 10 locations and nailed one card with aphids and one with seeds to the ground (“ground level”), and stapled one card with moth eggs and one with aphids to the top of a crop plant (“crop level”). Aphids were exposed for 24 h to avoid necrophagia, and other sentinel preys were exposed for 96 h. Predation measures took place twice, at the end of April 2016 and the end of May 2016 (see further details in Supplementary Material, Fig. S2). We then calculated for each type of prey the average predation rate per field across the 10 locations and the two periods.

We combined these two sets of data to assess the average predation rate at the landscape level. To do so, we first scaled predation rates between 0 and 1 and the average predation rate at the crop field level, i.e. the average across the four sentinel prey cards. Then, we calculated the average predation rate at the landscape level by calculating the average between the predation rate in woodland and the predation rate in the crop field.

### Statistical analysis

We carried out statistical analysis on three sets of data, two at the ecosystem-level: (i) in 30 woodland patches, (ii) in 30 cereal fields and one at the landscape-level (iii) in the 15 landscapes. In addition, we compared bird community composition from each ecosystem by using a principal component analysis (PCA) and by calculating beta diversity (see details in Supplementary Material, Fig. S4).

First, we tested the effect of land cover variables (i.e. proportions of woodlands and permanent grasslands, and crop diversity), local management intensity and plant diversity on the two sets of biocontrol proxies: (i) bird and carabid community metrics (i.e. abundance, Shannon index and Shannon index of insectivorous birds) and (ii) predation rate at ecosystem-level. We used generalized linear models (GLMs, *glmTMB* package,^86^) to create the full model; independent variables were standardized and, to avoid collinearity, we removed variables with variation inflation factor greater than 3 (*corvif* function,^87^). We used a Poisson distribution when the response variable was a count variable (e.g. abundance), or a negative binomial distribution if overdispersion was detected. Otherwise, response variables were modelled using Gaussian distributions. Then, we ran the *dredge* function (*MuMin* package,^88^) with a maximum of four independent variables in the same model to avoid model over parametrization and we selected the best model, i.e. with the lower AICc^89^. Distance-dependence in all model residuals was assessed using Moran’s I test (*Dharma* package^90^) and appeared to be not spatially related (all p > 0.05). We only found significant spatial autocorrelation of model residuals for the total abundance of birds in cereal fields. Then, we added GPS coordinates as fixed factors in the full model and show model results including spatial coordinates (Moran’s I test on residuals was then non-significant).

Second, at the landscape level, we assessed the effects of land cover variables (i.e. proportions of woodlands and permanent grasslands, and crop diversity) as well as the effect of landscape heterogeneity and the average management intensity on the two-biocontrol proxies: (i) bird community metrics and (ii) the average predation rate. We checked that these independent variables were not correlated (|ρ|< 0.7; Fig. S5). We also used the *dredge* function and we selected the best model. All analyses were performed with R software version 4.2.2.^91^.

### Regulations compliance

All methods were performed in accordance with the legislation. We did not collect plant samples and experiment on birds.

## Results

### Biological pest control potential in woodlands

#### Bird community metrics

**Figure 2:**
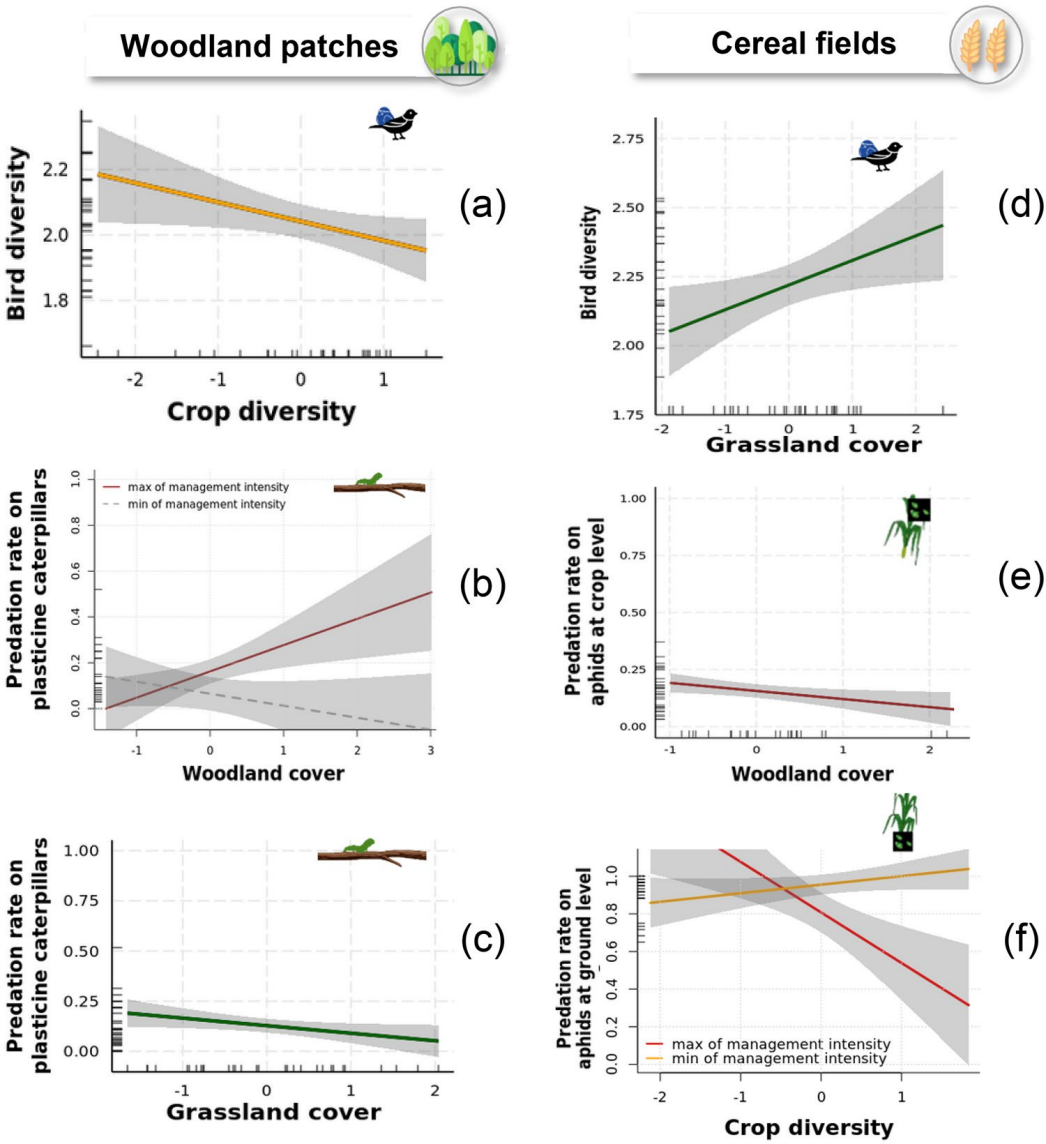
(**a**–**c**) Predicted responses of bird diversity and caterpillar predation in relation to crop diversity, woodland cover and grassland cover within woodland patches. (**d**–**f**) Predicted responses of bird diversity and aphid predation (crop- and ground-level) in relation to grassland cover, woodland cover and crop diversity. Model predictions from GLMs and associated 95% confidence intervals are represented by the solid lines and shaded areas. Dashed line correspond to the non-significant part of the interaction relationship.

The total abundance of birds in woodland patches was on average 19 individuals (± 3) and the species richness was 13 (± 2). The average Shannon diversity index was 2.04 (± 0.16) for the whole bird community, and 1.89 (± 0.20) when considering only insectivorous species. Crop diversity had a significant and negative effect on total bird diversity and the diversity of insectivorous birds, whereas the proportion of permanent grasslands had a significant positive effect on total bird diversity only (Tables 1, S3, Fig. 2). The best model to explain the total abundance of birds was the null model.

**Table 1:**
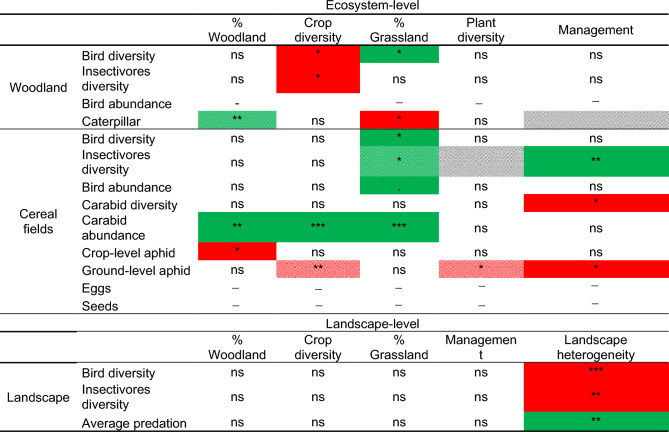
Main results of the effects of land cover variables, management intensity and local plant diversity on community indices and predation rates in woodland patches, in cereal fields and at landscape level.

Plant diversity in woodland patches corresponds to tree diversity and weed diversity in cereal fields. Management corresponds to the management intensity variable calculated in woodland patches; it corresponds to the total Treatment Frequency Index (TFI) in cereal fields and an average of both at landscape level.

Green boxes represent positive significant effects, red boxes negative significant effects from GLM analyses. Stripes indicate significant effects of interactions of two variables: stripes from left to right (///) correspond to positive interactions and stripes from right to left (\\\) correspond to negative interactions. White boxes with a dash represent null model as best model for the response variable. Empty boxes indicate that the variable was untested.

Non-significant effect: ns, *p < 0.05; **p < 0.01 and ***p < 0.001.

#### Caterpillar predation rates

The predation rate within woodland patches, i.e. the rate of plasticine caterpillars with predation marks, ranged between 0 and 0.52, with an average predation rate of 0.13. Woodland cover had a significantly positive effect on this predation rate. The interaction between woodland cover and management intensity had a significant positive effect on the predation rate, suggesting that higher woodland cover favoured higher predation rate when management intensity was high (Tables 1, S2, Fig. 2). The proportion of grasslands within the landscape also significantly and negatively influenced the predation rate (Table 1, Fig. 2).

### Biological pest control potential in cereal fields

#### Bird and carabid community metrics

Bird abundance in cereal fields was on average 13 individuals (± 4). Species richness was on average 10 (± 2). The average Shannon diversity index was 2.22 (± 0.22) for the whole bird community and 1.49 (± 0.23) when considering only insectivorous species. The proportion of permanent grasslands had a significant and positive effect on all bird community metrics, i.e. diversity of birds, insectivorous birds and marginally total abundance. The insectivorous bird diversity was also significantly and positively influenced by the interaction between the proportion of permanent grasslands and the local weed diversity, which suggests that the proportion of permanent grasslands increased insectivorous bird diversity when plant diversity was high. Moreover, local management intensity had a significantly positive effect on the diversity of insectivores (Tables 1, S4, Fig. 2).

Carabid abundance was on average 60 individuals and ranged between 10 and 188 individuals. Carabid diversity was on average 1.25 (± 0.47) and species richness was on average 7 (± 3). Woodland and grassland covers, as well as crop diversity had significantly positive effects on carabid abundance. In addition, local management intensity had a significantly negative effect on carabid diversity (Tables 1, S4, Fig. 2).

### Card predation rates

The predation rate in cereal fields was on average highest for ground-level aphids (0.95 ± 0.10), followed by seeds (0.55 ± 0.20) and moth eggs (0.45 ± 0.15). The lowest rate was found for crop-level aphids (0.15 ± 0.09). Correlations between the four types of prey cards ranged between 0.12 and 0.55 (Fig. S6). Woodland cover had a significantly negative effect on the predation rate of crop-level aphids. Moreover, local weed diversity, crop diversity and the interaction between crop diversity and local management intensity had a significantly negative effect on the predation rate of ground-level aphids. This interaction indicates that crop diversity had a significant and negative effect on the predation rate of ground-level aphids when local management intensity was high (Tables 1, S4, and Fig. 2).

### Biological pest control potential at landscape-level

#### Bird community metrics

**Figure 3:**
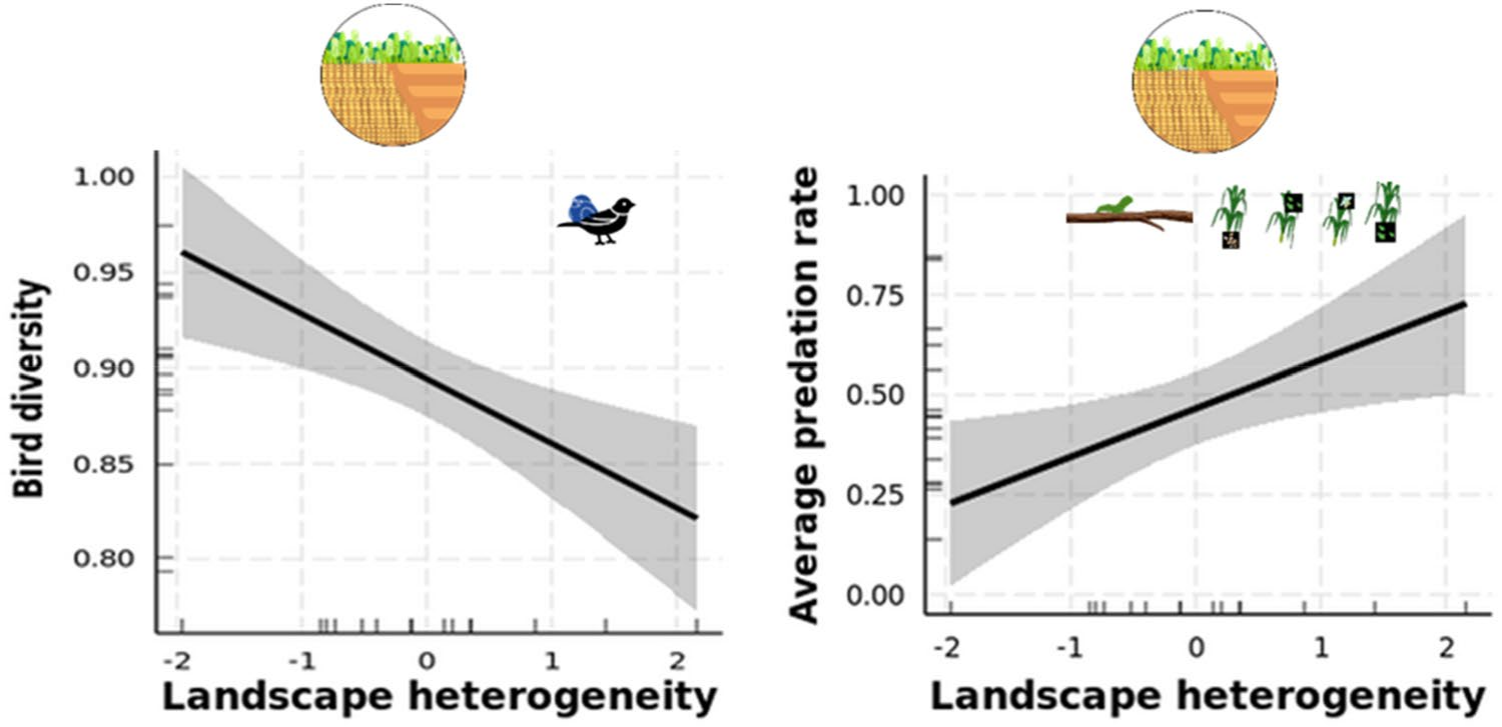
Predicted responses of bird diversity and average predation rate in relation to landscape heterogeneity. Model predictions from GLMs and associated 95% confidence intervals are represented by the solid lines and shaded areas.

The estimated bird abundance at landscape-level was on average 20 individuals and species richness was on average 13 (± 1). The average Shannon diversity index was 2.45 (± 0.12) for the whole community and 1.88 (± 0.17) when considering only insectivorous species. Landscape heterogeneity had a significant and negative effect on the Shannon diversity index of the whole bird community and those of insectivorous bird species. Woodland, grassland cover and crop diversity had no significant effect on bird community metrics. In addition, average management intensity did not influence bird community metrics at landscape level (Table 1, Fig. 3).

### Average predation rate

The predation rate at landscape-level was on average 0.44 and ranged from 0.31 to 0.58 (Figs. S7, S8). Landscape heterogeneity had a significant and positive effect on the average predation rate. Neither land cover variables nor management intensity had significant effects on average predation rate (Tables 1, S5, Fig. 3).

## Discussion

We analyse the effects of land cover variables on the biological control potential of both forest and agricultural pests at the ecosystem and landscape levels. Our analysis at the ecosystem level shows that land cover variables, in particular cover proportion of different semi-natural habitats, do influence biological pest control potential both in cereal fields and woodland patches, and have antagonistic effects in the two ecosystems. In particular, the proportion of woody semi-natural habitats favours caterpillar predation in woodlands but reduces crop-level aphid predation in cereal fields. Our analysis at the landscape level then shows that increasing landscape heterogeneity represents a valuable solution to manage trade-offs between ecosystems and favours the average predation rate across these ecosystems. Our study however shows that results strongly vary depending on the proxy of biological control potential considered and depending on the local context, in particular land use intensity. Further studies are needed to more finely assess the effect of landscape structure on biological control across ecosystems.

### Land cover variables have opposite effects in woodlands and cereal fields

Our first main result is that land cover variables influence biological pest control potential both in cereal fields and woodland patches, and have opposite effects in woodlands and cereal fields. Woodland cover increases predation rates on caterpillars in woodland patches whereas it decreases predation rates on crop-level aphids in cereal fields. Crop diversity reduces bird diversity in woodlands whereas it promotes carabid abundance in cereal fields. Permanent grasslands are also important semi-natural habitats in our study site and their proportion reduces caterpillar predation in woodlands whereas it increases bird and carabid abundances in cereal fields.

First, our results confirm the beneficial effect of woodland cover on caterpillar predation rates in woodland patches^92,93^, especially when woodland management intensity is higher. However, they do not confirm its beneficial effect on predation rates of phytophagous insects and weed seeds in cereal fields. Although we did observe a positive effect of woodland cover on carabid abundance, the predation of crop-level aphids in cereal fields decreased with woodland cover. This result partially contrasts with previous studies highlighting that complex agricultural landscapes, i.e. with a high proportion of cover of semi-natural habitats, enhance the abundance and diversity of natural enemies and may therefore increase pest control in crop fields^15,16,23^. Particularly, in our study, we did not consider the different types of semi natural-habitats, such as hedgerows, grassy strips, rivers, which can favour natural enemies communities and increase biological pest control^94,95^. Here we focused on woodlands^21–23^. A possible explanation for the opposite effect of woodland cover on carabid abundance and crop-level aphid predation rates may be that only few carabid species, carrying out their entire life cycle in crop fields, contribute to predation within conventional cereal fields^96^. Our result suggest that woodland cover may decrease the diversity or abundance of these predator species that contribute to crop-level aphid predation within cereal fields^97^.

Second, our results confirm the beneficial effect of crop diversity on carabid abundance in cereal fields. This result is consistent with the resource complementarity hypothesis, i.e. the positive effect of different land cover types on biodiversity^18^. This positive effect is associated with a positive effect on predation rate but is modulated by pesticide use. Indeed, we observed a positive effect of crop diversity on ground-level aphid predation when pesticide use is low, whereas it turns negative when pesticide use is high. This result is consistent with previous studies^30,98,99^ suggesting that species favoured by crop diversity contribute to ground-level aphid predation when management intensity is low (i.e. spill over process). However, when management intensity is high, ground-level aphid predation is more likely to be provided by a few species adapted to cereal fields and higher management intensity^96^. We also observed a negative effect of crop diversity on bird diversity in woodland patches. Our results on bird community composition showed that community differed between cereal fields and woodlands and this was mainly related to species abundance variation (i.e. individual replacement from one species to another). Indeed, although some habitat-specialist species were counted in woodland patches, we also observed many habitat-generalist species in both ecosystems. This result confirms that the diversity of the landscape “matrix” does influence biodiversity and ecosystem processes occurring within woodland patches^50,100^ and that semi-natural patches are necessary to maintain some other bird species^101^.

Finally, our results confirm the beneficial effect of permanent grasslands on carabid and bird abundance in cereal fields, as well as on bird diversity in cereal fields and woodland patches. This result is consistent with the fact that semi-natural habitats enhance the abundance and diversity of natural enemies in agricultural landscapes^15,16,23^. This result also suggests that the presence of permanent grasslands in the agricultural matrix has beneficial effects on bird diversity in woodland patches, i.e. that some bird species benefit from the complementation between grassland and woodland and also that it may improve connectivity between small woodland patches^102–104^. However, our results show that permanent grasslands decrease biological control in woodlands. A possible explanation could be that biological pest control in forests is mainly done by forest-dependent species^13^, which are not favoured by adjacent grasslands and may be reduced by increased competition with multi-habitat species.

Our analysis at the ecosystem level shows that land cover variables do influence differently biological pest control potential in cereal fields and woodland patches. In order to search for potential management compromises, a way seems to move to a higher level through a landscape approach integrating these different ecosystems.

### Increasing landscape heterogeneity increases the average predation rate at the landscape level

Our second main result is that increasing landscape heterogeneity enhances the average predation rate across woodland patches (caterpillar predation) and cereal fields (predation of aphids, moth eggs and weed seeds). This result is consistent with those at the ecosystem level, suggesting that landscape heterogeneity promotes a diversity of predator species contributing to predation respectively in woodlands and cereal fields^50–52^. Our study therefore shows that increasing landscape heterogeneity, through the proportion of semi-natural habitats in particular, represents a valuable lever to sustain biological pest control in both crop fields and woodlands. As cereal fields and woodland patches are associated with different food webs and pest issues we used standardized protocols adapted to assess biological pest control within each ecosystem. Then, in the present study, we calculated the average predation rate between woodland patches and cereal fields, i.e. we gave the same weight to biological pest control in each ecosystem. Nevertheless, we are aware that pests may cause higher damages, or may trigger higher financial losses in cereal fields than in woodland patches^105,106^. When this is the case, it may be necessary to attribute different weights to predation rates in different ecosystems. Further studies should therefore assess the level of demand in predation rates from different stakeholders for different ecosystems in order to better identify which landscape management strategies are the most adapted^107^.

We observed a negative effect of landscape heterogeneity on bird diversity at the landscape level. Although this result may seem surprising at first, it is actually consistent with the fact that semi-natural habitats and agricultural habitats host different proportions of farmland biodiversity. Indeed, in Europe, 49% of all species are unique to semi-natural habitats (woodland, hedgerows, and extensively managed grasslands) whereas only 26% are unique to crop fields and intensively managed grasslands, and the remaining 25% occur in the two types of habitats^108^. In our study area, landscape heterogeneity is negatively correlated with the proportion of permanent grasslands (|ρ| = − 0.6), which are mostly extensively managed. Although the present study did not aim to assess biodiversity per se and considered bird communities as a proxy for biological control, the antagonistic effect of landscape heterogeneity on predation rate and bird diversity suggests that there may be a trade-off between biological control and biodiversity conservation^109^. Overall, our study confirms the relevance of studying multiple ecosystems and multiple components of biological control simultaneously. Future studies should further explore the role of landscape heterogeneity on other taxonomic groups, such as parasitoids, contributing to biological control in woodlands and cereal fields.

### Local context modulates land cover effects on biological control

Our third main result is that local management intensity and local plant diversity have significant effects on biological pest control potential, and that these effects modulate the effects of land cover variables, in both woodland patches and cereal fields.

In woodland patches, local management intensity had a positive effect on predation rate and increased the effect of woodland cover on predation rate, i.e. woodland cover had a more positive effect when local management intensity was high. This suggests that in these farm woodlands, which are not very mature (e.g. few cavity trees) and whose management intensity is rather low (i.e. cuts for firewood, some for timber) and variable, increasing woodland cover can provide more resources (i.e. supplementation process) and enhance pest predation^13^. Surprisingly, we found no significant effect of tree diversity on either predation rate or bird diversity in woodland patches. This result contrasts with the hypothesis that heterospecific neighbours favour greater abundance and diversity of natural enemies because of the availability of complementary resources^19^. However, a meta-analysis recently showed that this hypothesis may only apply to generalist predator species, and not to specialists^110^. In our study area, although woodlands are ancient, they are not mature, i.e. they are mostly composed of young trees, and are likely to host more generalist predator species^111^.

In cereal fields, local plant diversity had a negative effect on ground-level aphid predation whereas it increased the effect of permanent grassland on the diversity of insectivorous birds, i.e. permanent grassland had a more positive effect when local plant diversity was high, whereas it decreased ground-level predation rate. These results are consistent with previous studies showing that local plant diversity can have contrasted effects: beneficial on the abundance and diversity of generalist predators and negative or neutral on herbivores and specialist predators^45^. Our results suggest that plant diversity may negatively affect specialist predators of aphids in cereal fields but have a positive effect, in combination with permanent grassland, on generalist bird species. In addition, our results showed that the local management intensity decreased the effect of crop diversity on the predation rate on ground-level aphids. These results are in line with previous studies showing that the intensity of farming practices modulates the effects of landscape heterogeneity on biological pest control^5,30,112^. Our results also show that the local management intensity decreased the diversity of carabids whereas it increased the diversity of insectivorous birds in cereal fields. These results are consistent with the fact that higher farming intensity may favour generalist species able to use and subsist in these fields^96^. Similarly, bird communities are affected by both landscape structure and local context^39,113^. For instance, Barbaro et al.^114^, showed that organic management in vineyards, especially, grass cover strategy combined with landscape heterogeneity increased functional bird diversity.

## Conclusion

While a growing number of studies have highlighted the role of landscape structure on biological pest control in crop fields^5,15,16^, no study has so far investigated its effects on biological pest control in woodland patches and crop fields jointly. Our study shows that landscape structure influences biological pest control, both in cereal fields and woodland patches, and have antagonistic effects in the two ecosystems with potential biological control that were specific to them. It also shows that landscape heterogeneity favours landscape-level predation rates. This study therefore contributes to the increasing body of literature showing that landscape-level management is necessary to achieve agroecological transition. Our work provides a valuable contribution by showing that: (i) landscape-level management may have antagonist effects on biological pest control in different ecosystems and (ii) local management practices modulate the effects of landscape-level management in these ecosystems. Overall, our study confirms the need for integrative studies, i.e. considering simultaneously multiple ecosystems and multiple response variables, in order to identify potential trade-offs. This study also raises questions about the diversity of biological control in different ecosystems and whether they should be prioritised or at least how they can be considered together in landscape management strategies. Moreover, it confirms the value of conducting studies at multiple levels, in particular ecosystem and landscape levels, in order to identify solutions to manage trade-offs in nature-based solutions.

### Supplementary Information

Supplementary Information.

## Data Availability

The datasets generated and/or analysed during the current study are available in the DataINRAE repository, 10.1016/j.agee.2021.107810.
