# Supplementary material for: Increasing landscape heterogeneity as a win–win solution to manage trade-offs in biological control of crop and woodland pests
Source: Sci Rep. 2023 Aug 21;13:13573. doi: 10.1038/s41598-023-40473-2 (PMC10442452; doi:10.1038/s41598-023-40473-2)
Supplement: Supplementary file 1 — Supplementary Information. [file 41598_2023_40473_MOESM1_ESM.docx]

# Supplementary Material

## Appendix A: Description of the factors used to characterize management intensity in woodland patches

Table S 1: The ten factors of the Index of Biodiversity Potential (IBP, 1) were calculated in woodland patches and are known to be favourable to forest biodiversity. Factor A to G are associated with forestry management. Factor H to J are associated with context. We used four factors (C, D, E and F) to calculate management intensity in our woodlands. For each factor, a score is attributed 0, 1, 2 or 5, then IBP is the sum of the ten scores.

|  | | Expected effect of repeated harvesting operations in coppice with standards | |
| --- | --- | --- | --- |
| Factor | Definition | Score increase | Score decrease |
| **C** | Large dead wood on the ground | Never since the time needed to get such feature is much longer than the time between two cuts (2) | Yes since owners are not used to leave biological legacies |
| **D** | Large standing dead wood | Never since the time needed to get such feature is much longer than the time between two cuts (2) | Yes since owners are not used to leave biological legacies |
| **E** | Very large live trees | Almost never since the maximum cutting commercial diameter for standards is generally less than 70 cm | Yes since owners are not used to leave biological legacies |
| **F** | Micro-habitats bearing trees | Never since the time needed to get such feature is much longer than the time between two cuts (3) | Yes since owners are not used to leave biological legacies |
| Other factors not used in our characterization of management intensity | | | |
| **A** | Native species | Yes, after a few years of regrowth (more regular presence of pioneer and post-pioneer species) | Possible, if a non-native tree species thrive and competes strongly with native ones (e.g. *Robinia pseudoacacia*) |
| **B** | Vertical structure of the vegetation | Possible after a few years of regrowth | Yes, just after harvesting |
| **G** | Openness | Yes, but often capped at score 2 | Sometimes, when the initial stand before cutting was very textured |
| Other factors not used in our characterization of management intensity and related to context | | | |
| **H** | Woodland continuity over time |  |  |
| **I** | Aquatic habitats |  |  |
| **J** | Rocky habitats |  |  |

## Appendix B: Estimation of predation rate in woodlands

In each woodland, 3 to 5 plots of about 26 m radius were chosen according to the woodland surface. Plasticine caterpillars were attached to a thin metal wire around branches of 3 shrubs/trees within each one of the three plots. Shrubs and trees belong to one of the following five species: *Crataegus monogyna, Sorbus torminalis*, *Prunus avium*, *Carpinus betulus, Corylus avellana*. On each shrub/tree, we placed 3 plasticine caterpillars at different heights between 130 and 190 cm. Then, 27 to 45 plasticine caterpillars were placed per woodland patch.


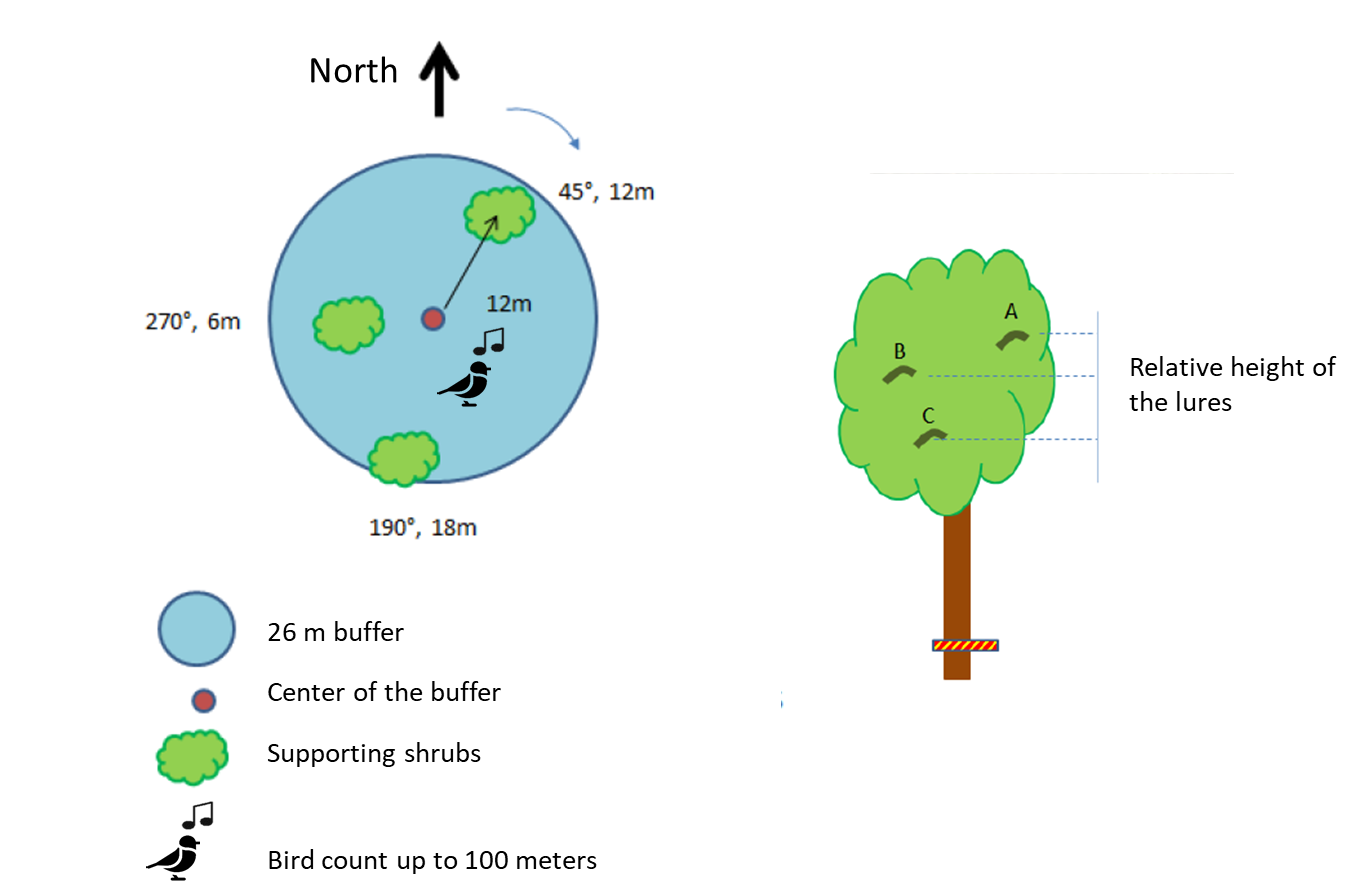


Figure S 1: Sampling design of ecological measurements in woodlands.

## Appendix C: Estimation of predation rate in cereal fields

We selected four types of pest according to their diversity, their similarity to winter cereal pests and the diversity of targeted natural enemies, while considering the constraints of rearing (4,5).

Four sentinel prey cards were positioned in 10 plots evenly distributed along two parallel 50 m long transects. The transects were perpendicular to the field border, with the first card placed 50 m away from the border and the last 100 m away.

The four complementary types of predation were measured with three prey species glued to 5 x 5 cm sandpaper cards. The glues were chosen among a set of low toxic glues after practical tests to ensure the prey were just fixed but not mired and that they would not come unstuck during the period of exposure. Insect predation was assessed using predation cards on which three adult pea aphids *Acyrthosiphon pisum* were glued (glue: UHU® Twist & Glue solvent free). The cards were placed both on the ground (between 10 to 20 cm apart) and to the top of a crop plant, just under inflorescence. In addition to aphids, predation cards containing clusters of *Ephestia kuehniella* (Lepidoptera) eggs were placed at the top of a crop plant. *Ephestia* eggs are too small to allow precise enumeration, so a 5 mm-wide cluster was glued to the card (glue: SADER® all-purpose solvent-free). Seed predation was assessed using *Viola arvensis* seeds (ten per card, glue: SADER® WOOD PRO D3 diluted with two-thirds of water) exposed on the ground. The prey species exposed were selected according to those used in international devices (e. g. 5). They were chosen according to their diversity, their similarity to winter crop pests and the diversity of targeted natural enemies, while considering the constraints of rearing (4,5). In addition, these prey types are representative of groups responsible for the main damage in crop fields and allow assessing the action of the generalist predators.

The number of prey that remains on the cards at the end of the period of exposure was counted in the field, except for *Ephestia* predation, which, because of their small size, were counted using a magnifying binocular in the laboratory. Two classes were used for *Ephestia* predation: unconsumed (less than 5% of the eggs missing) or consumed (more than 5%).


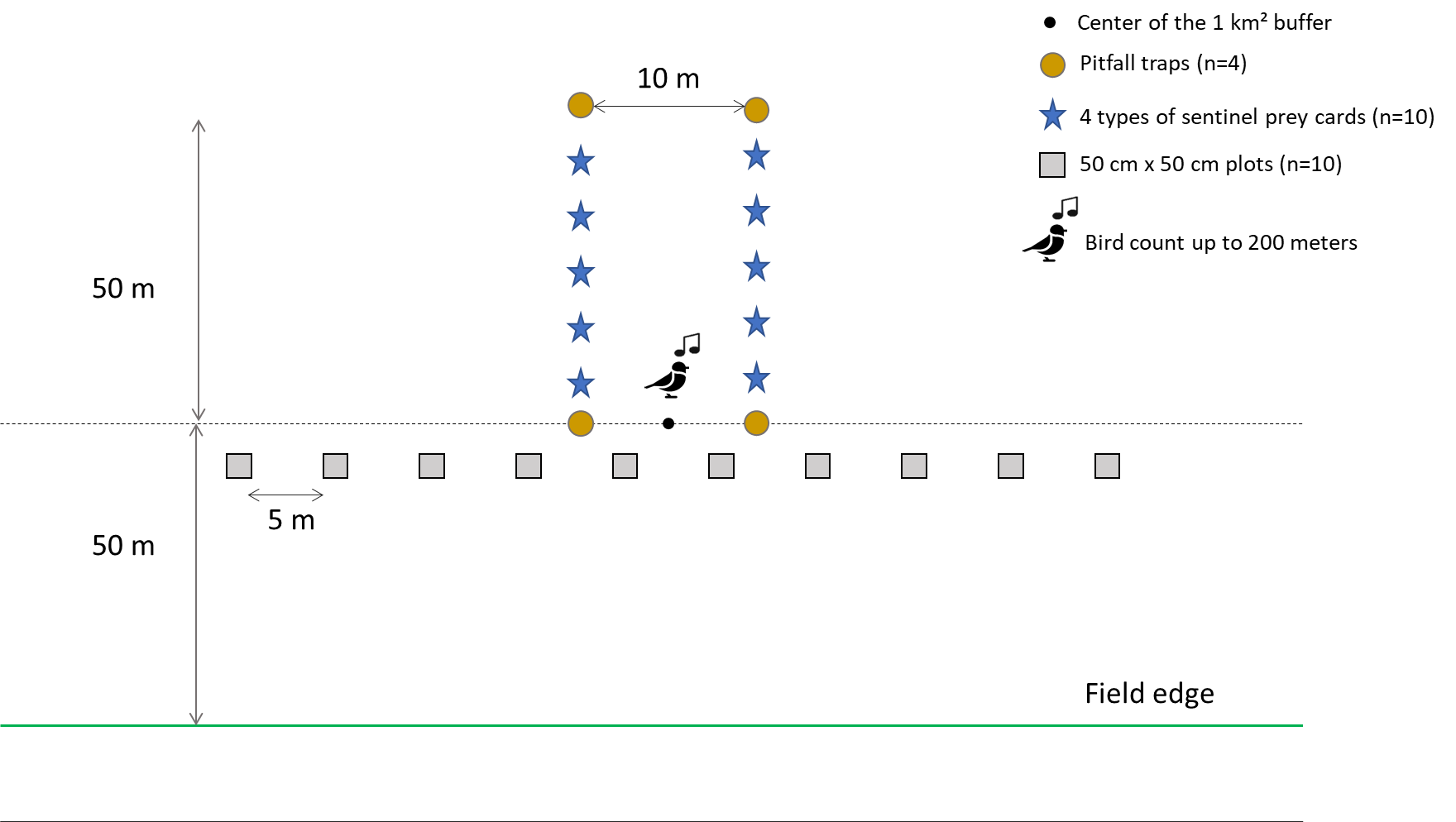


Figure S 2: Sampling design of ecological measurements in cereal fields

## Appendix D: Description of variables

Table S 2: Distribution of the landscape metrics, local context variables and ecological measurements in each habitat.

|  |  | Woodlands | Cereal fields | Landscape level |
| --- | --- | --- | --- | --- |
|  | Variable name | Mean [min,max] | Mean [min,max] | Mean [min,max] |
| Landscape metrics | Proportion of woodland cover | 15.8 [2.98; 46.1] | 13.1 [0.48; 38.9] | 12.3 [2.63; 24.5] |
|  | Proportion of permanent grassland cover | 28.9 [8.61; 53.9] | 21.2 [0.30; 47.9] | 55.5 [12.7; 87.9] |
|  | Crop diversity | 0.88 [0.43; 1.17] | 0.94 [0.52; 1.25] | 0.90 [0.46; 1.17] |
|  | Landscape heterogeneity | 1.38 [1.01; 1.57] | 1.37 [0.82; 1.58] | 1.26 [0.93; 1.62] |
| Local management intensity and plant diversity | Diversity of trees within the woodlands | 0.7 [0; 1.26] |  |  |
|  | Index of potential biodiversity directly related to management practices  *IBP_C_****_-F_*** | 7 [2; 17] |  |  |
|  | Management intensity in the woodlands | 0.14 [0.06; 0.5] |  |  |
|  | Plant diversity |  | 0.79 [0.04; 1.72] |  |
|  | Total of treatment frequency index |  | 4.41 [1.84; 10.1] |  |
|  | Management intensity at landscape level |  |  | 3.97 [2.04; 5.86] |
| Predation rates | Predation rate on plasticine caterpillars | 0.13 [0; 0.52] |  | 0.12 [0; 0.31] |
|  | Predation rate on aphids at the crop level |  | 0.15 [0.03; 0.36] | 0.15 [0.05; 0.28] |
|  | Predation rate on aphids at the ground level |  | 0.95 [0.65; 1] | 0.92 [0.71; 1] |
|  | Predation rate on *Ephestia* eggs at the crop level |  | 0.45 [0.20; 0.70] | 0.47 [0.20; 0.70] |
|  | Predation rate on weed seeds at the ground level |  | 0.55 [0.24; 0.89] | 0.55 [0.24; 0.89] |
|  | Predation rate averaged on all prey type |  |  | 0.44 [0.31; 0.58] |
| Bird community metrics | Shannon diversity of birds | 2.04 [1.66; 2.35] | 2.23 [1.73; 2.56] | 2.45 [2.21; 2.65] |
|  | Shannon diversity of insectivorous birds | 1.89 [1.33; 2.21] | 1.49 [1.06; 2.02] | 1.88 [1.46; 2.14] |
|  | Total abundance of birds | 19 [12; 23] | 13 [8; 23] | 15 [20; 24] |
| Carabids community metrics | Shannon diversity of carabids |  | 1.25 [0.23; 2.27] |  |
|  | Total abundance of carabids |  | 60 [10; 188] |  |

Figure S 3: Representation of the different scales used and associated measurements in our study. (A) Measurements at the ecosystem-level; land cover metrics were calculated within a 500m buffer radius. (B) Measurements at the landscape-level; landscape heterogeneity was assessed within the fusion of both ecosystems buffers; the management intensity and predation rates were averaged.


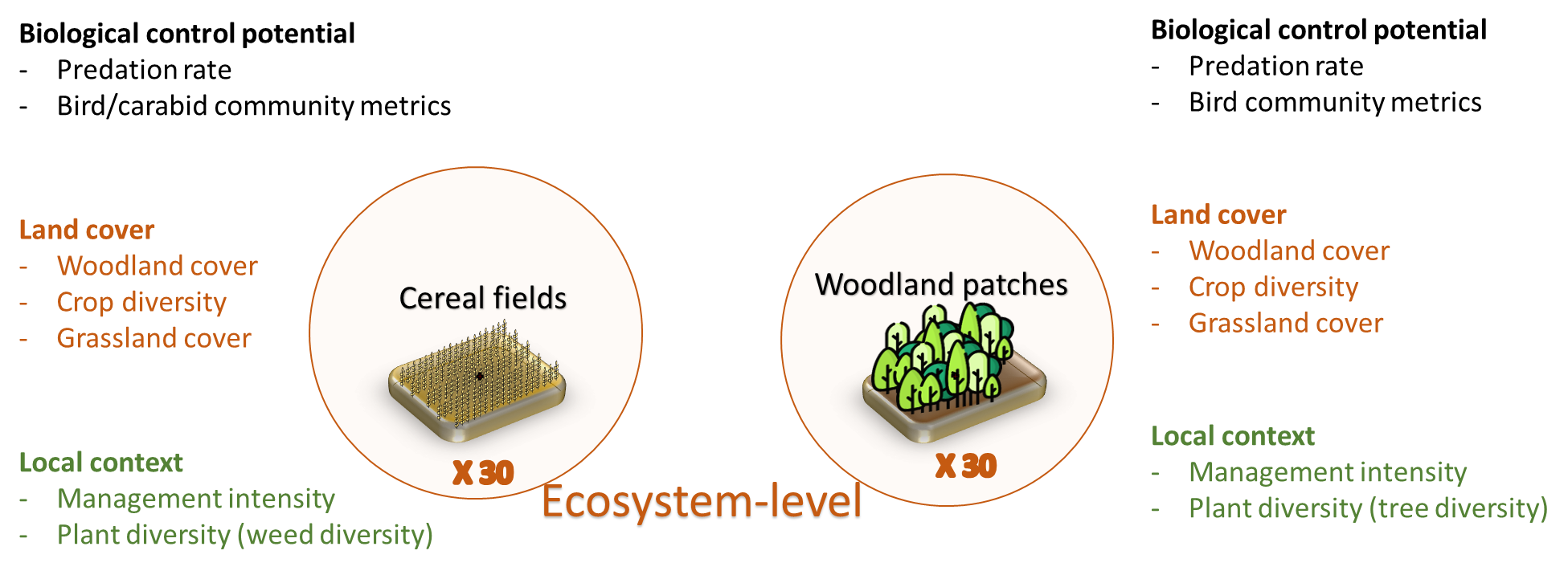

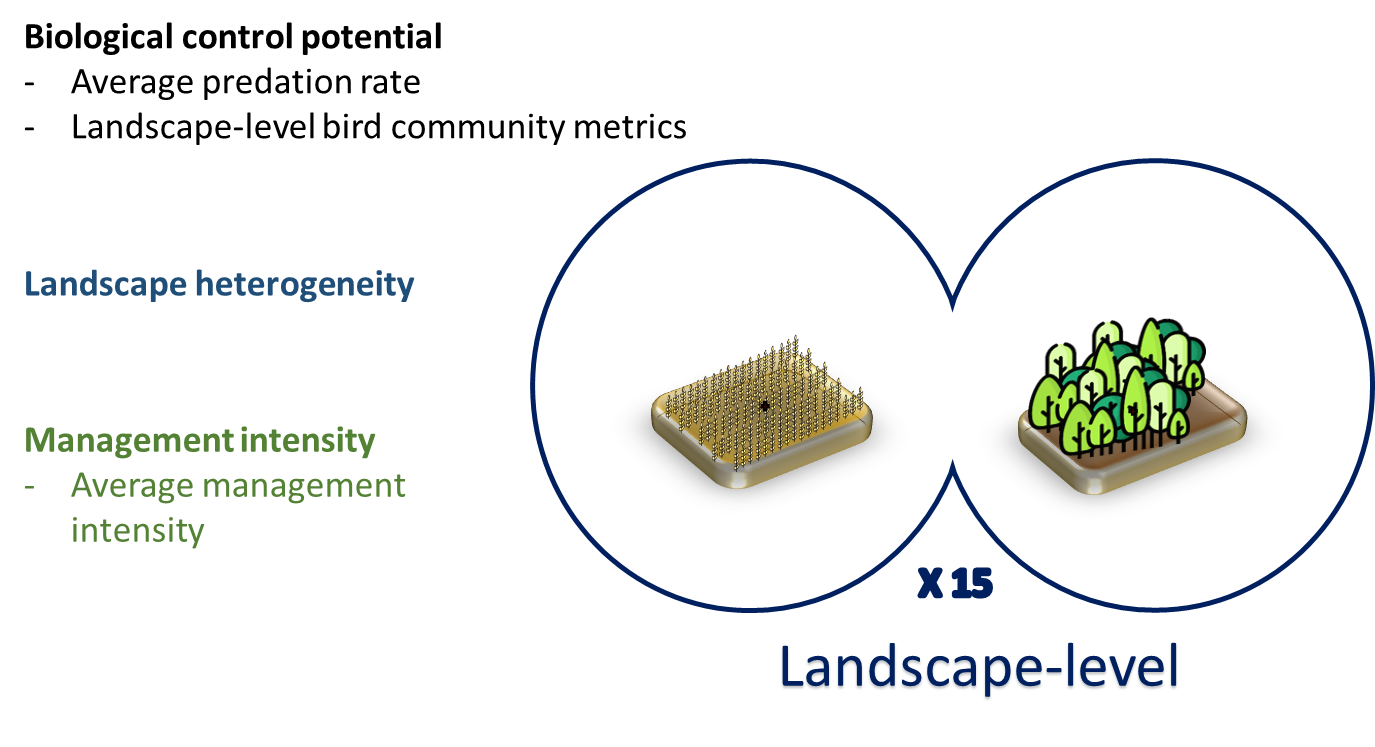


(A)

(B)

## Appendix E: Comparison between bird communities in woodlands and bird communities in cereal fields

We recorded 562 individuals within the 30 woodland patches through the two sampling periods, that belonged to 31 species. The assemblage of the bird community was composed for the first four major species with: *Sylvia atricapilla* (12.6% of the total abundance), *Erithacus rubecula* (9.8%), *Phylloscopus collybita* (9.6%) and *Parus major* (9%).

We recorded 391 individuals within the 30 cereal fields that belonged to 39 species. Here, bird community is rather more associated with the crop mosaic than the cereal field itself. The most four frequent species were *Sylvia atricapilla* (11.5% of the total abundance), *Turdus merula* (10.7%), *Luscinia megarhynchos* (6.9%) and *Parus major* (6.1%).

We compared bird community composition from each ecosystem with beta diversity. Using the *betapart* package (6) we calculated total dissimilarity between both ecosystems with Bray’s index β*_BC_*, which can be described as the amount of abundance that is shared by the two assemblages. We divided beta diversity into two components (7): balanced variation (β*_BC_*_._*_BAL_*) and abundance gradient (β*_BC_*_._*_GRA_*). The β*_BC_*_._*_BAL_* component illustrates perfectly balanced variation in species abundance, i.e. the increase in abundance of some species is exactly the same as the decrease in abundance of others. Whereas the β*_BC_*_._*_GRA_* shows that the abundance of all species decreases along a gradient between the sites compared. A principal component analysis (PCA) was also carried out with all the bird species between ecosystems.

Bird beta diversity between woodlands and cereal fields was 0.50 for β*_BC_* (β*_BC_*_._*_BAL_* = 0.39, β*_BC_*_._*_GRA_* = 0.11). The PCA on all species sampled in both woodlands and cereal fields showed different communities of bird between woodland patches and cereal fields (Figure S 7).


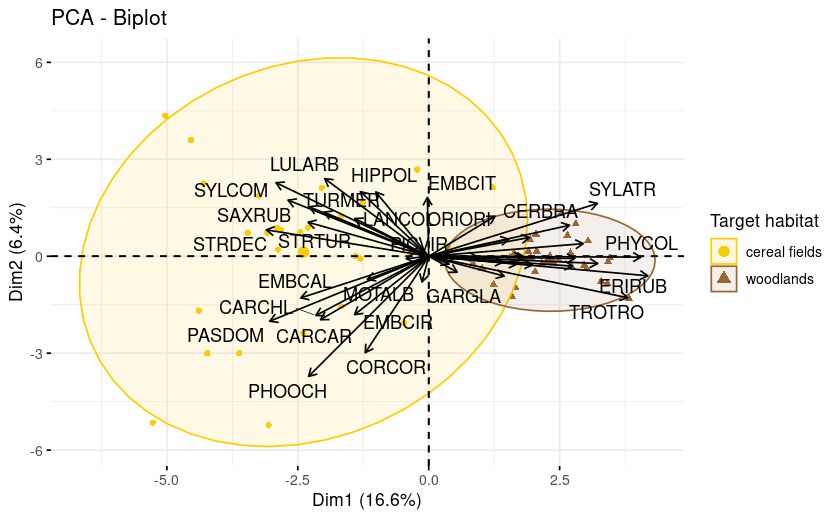


Figure S 4: Principal Component Analysis of sampled species within woodlands (N = 30) and cereal fields (N = 29). Species name are abbreviated (ALAARV: Alauda arvensis, ANTTRI: Anthus trivialis, CARCAR: Carduelis carduelis, CARCHL: Carduelis chloris, CERBRA: Certhia brachydactyla, COLPAL: Columbus palumbus, CORCOR: Corvus corone, CUCCAN: Cuculus canorus, CYACAE: Cyanistes caeruleus, DENMAJ: Dendrocopos major, DENMED: Dendrocopos medius, DENMIN: Dendrocopos minor, DRYMAR: Dryocopus martius, EMBCAL: Emberiza calandra, EMBCIR: Emberiza cirlus, EMBCIT: Emberiza citrinella, ERIRUB: Erithacus rubecula, FRICOE: Fringilla coelebs, GARGLA: Garrulus glandarius, HIPPOL: Hippolais polyglotta, LANCOL: Lanius collurio, LULARB: Lullula arborea, LUSMEG: Luscinia megarhynchos, MOTALB: Motacilla alba, ORIORI: Oriolus oriolus, PARMAJ: Parus major, PASDOM: Passer domesticus, PHOOCH: Phoenicurus ochruros, PHYBON: Phylloscopus bonelli, PHYCOL: Phylloscopus collybita, PICVIR: Picus viridis, REGIGN: Regulus ignicapilla, SAXRUB: Saxiocola rubicola, SITEUR: Sitta europaea, STRDEC: Streptopelia decaocto, STRTUR: Streptopelia turtur, STUVUL: Sturnus vulgaris, SYLATR: Sylvia atricapilla, SYLCOM: Sylvia communis, TROTRO: Troglodytes troglodytes, TURMER: Turdus merula, TURPHI: Turdus philomelos, TURVIS: Turdus viscivorus).

## Appendix F: Correlations between land cover metrics at landscape-level and among predation rates

Landscape heterogeneity

Woodland

Crop diversity


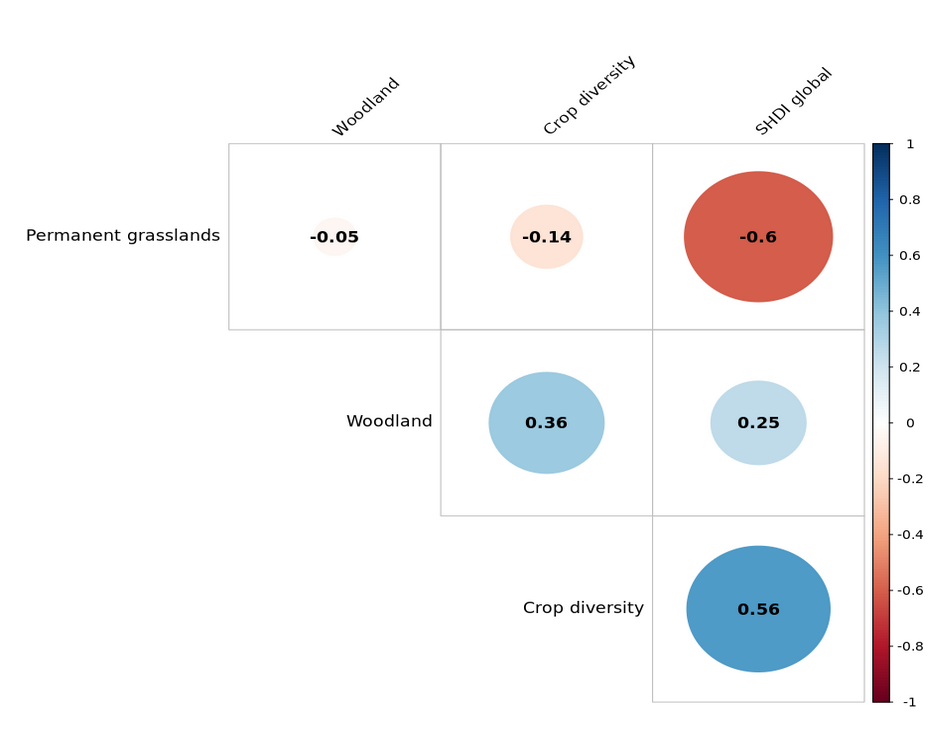


Figure S 5: Spearman’s correlations between landscape metrics at landscape level (N=15).

We looked at correlations among all measures of predations rates using Spearman’s correlations, first among the four sentinel prey, then among ecosystem-level predation rates measured in woodlands and cereal fields. Correlations among the four types of prey cards within crop fields ranged from 0.14 to 0.57 (Spearman’s rho; Figure S 5), thus confirming that these different types of prey provide complementary information. Correlations between predation rate on plasticine caterpillars in woodlands, and predation rate on the four types of prey cards in cereal fields ranged from -0.17 to 0.66 (Spearman’s rho; Figure S 7), thus suggesting that it may be difficult to have simultaneously high predation rates in woodlands and crop fields within a given landscape.


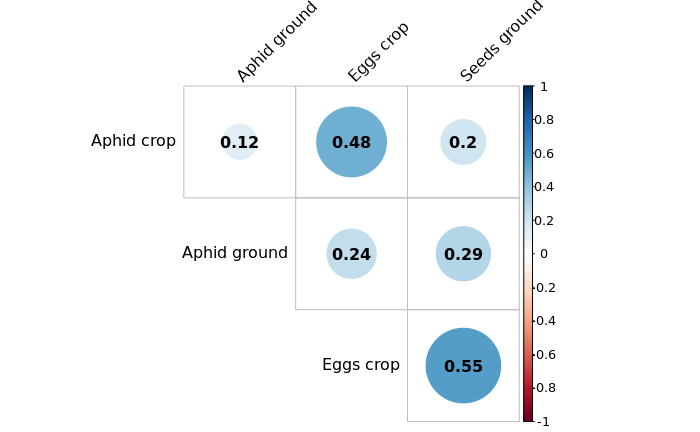


Figure S 6: Spearman’s correlations between predation rates in cereal fields (N = 30).


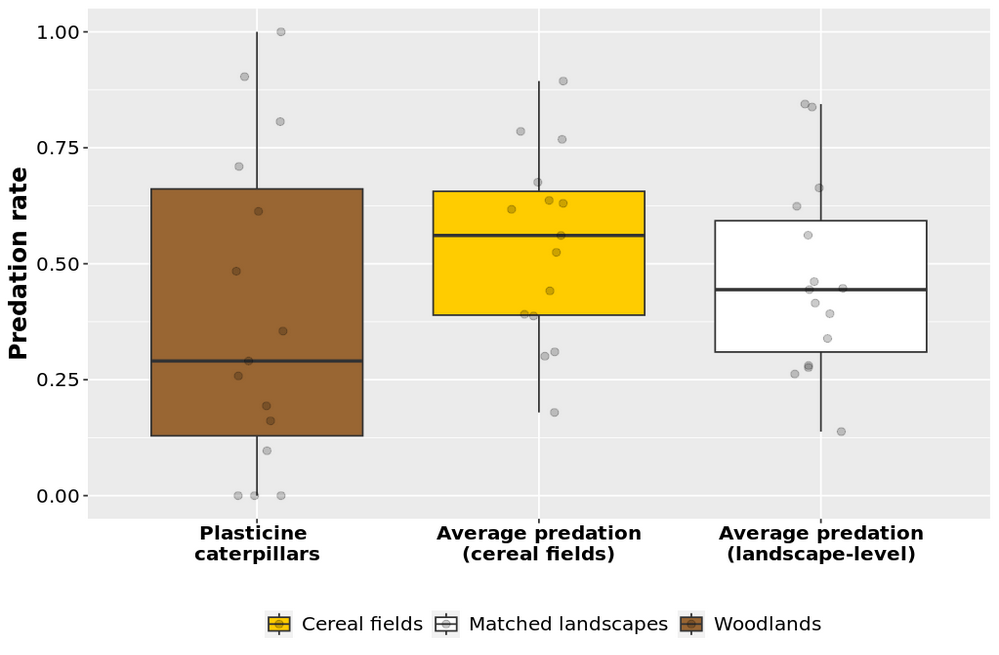


Figure S 7: Comparison of predation rates between ecosystems: brown boxplot corresponds to woodland patches in matched landscapes (N = 15), yellow boxplot corresponds to cereal fields in matched landscapes (N = 15) and white boxplot corresponds to the average predation rate (N = 15).


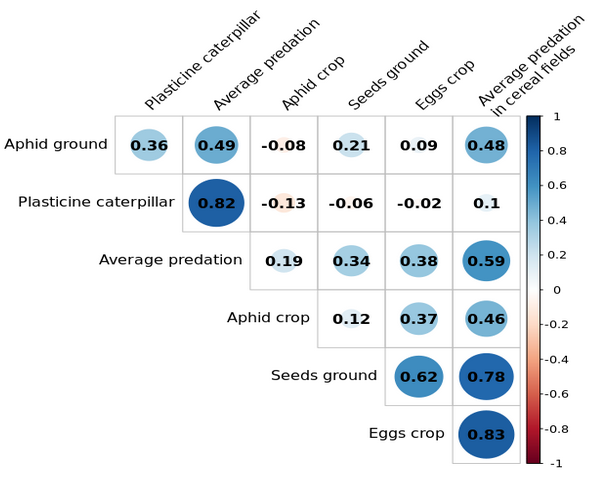


Figure S 8: Spearman’s correlations between predation rates measured in woodlands, cereal fields and averaged within the 15 landscapes (N = 15).

## Appendix G: Detailed results of the best models

Table S 3: Standardized estimates and standards errors (± SE) of the landscape and local metrics on biodiversity and predation rate at ecosystem level in woodland patches. Here the results of the best model (based on AICc criterion) are shown. The response variables not present in this section had a best model equal to the null model. * p < 0.05; ** p < 0.01 and *** p < 0.001**.**

| **Woodland patches (N = 30)** | | | | |
| --- | --- | --- | --- | --- |
| Formula: Response variable ~ (Woodland + Crop diversity + Permanent grassland) * (Managt intensity + Tree diversity) | | | | |
|  | **Response variable** | **Explanatory variables** | **Estimate (± SE)** | **P-value** |
| **BIRD COMMUNITY** | Shannon diversity of birds | **Permanent grassland**  **Crop diversity** | **0.0641 (±0.0253)**  **-0.0618 (±0.0253)** | **0.0113 ***  **0.0146 *** |
|  | Shannon diversity of insectivorous birds | **Crop diversity** | **-0.0764 (±0.0353)** | **0.0304 *** |
| **PREDATION RATE** | Predation on caterpillars | Managt intensity  **Permanent** **grassland**  **Woodland**  **Managt intensity:Woodland** | 0.0302 (±0.0171)  **-0.0376 (±0.0171)**  **0.0659 (±0.0229)**  **0.0978 (±0.040)** | 0.0767  **0.0279 ***  **0.00415 ****  **0.0146 *** |

Table S 4: Standardized estimates and standards errors (± SE) of the landscape and local metrics on biodiversity and predation rates at ecosystem level in cereal fields. Here the results of the best model (based on AICc criterion) are shown. The response variables not present in this section had a best model equal to the null model. * p < 0.05; ** p < 0.01 and *** p < 0.001**.**

| **Cereal fields (N = 30)** | | | | |
| --- | --- | --- | --- | --- |
| Formula : Response variable ~ (Woodland + Crop diversity + Permanent grassland) * (Managt intensity + Plant diversity) | | | | |
|  | **Response variable** | **Explicative variables** | **Estimate (± SE)** | **P-value** |
| **BIRD COMMUNITY** | Shannon diversity of birds | **Permanent grassland** | **0.0891 (±0.0369)** | **0.0159 *** |
|  | Shannon diversity of insectivorous birds | Plant diversity  **Permanent grassland**  **Managt Intensity**  **Plant diversity:Permanent grasslands** | -0.0414 (±0.0332)  **0.0726 (±0.0362)**  **0.108 (±0.0348)**  **0.0975 (±0.0377)** | 0.212  **0.0453 ***  **0.00188 ****  **0.00963 **** |
|  | Total abundance of birds ^1^ | Permanent grassland  **Yl93m** | 0.0978 (±0.0547)  **-0.153 (±0.0549)** | 0.0738 .  **0.0053 **** . |
| **CARABID COMMUNITY** | Shannon diversity of carabids | **Managt Intensity** | **-0.197 (±0.0784)** | **0.0119 *** |
|  | Total abundance of carabids | **Permanent grassland**  **Woodland**  **Crop diversity** | **0.404 (±0.119)**  **0.350 (±0.121)**  **0.435 (±0.122)** | **7.16e-04 *****  **0.0039 ****  **3.76e-04 ***** |
| **PREDATION RATE** | Predation on crop-level aphids | **Woodland** | **-0.0355 (±0.0148)** | **0.0163 *** |
|  | Predation on ground-level aphids | **Plant diversity**  **Crop diversity**  **Managt Intensity**  **Crop diversity:Managt Intensity** | **-0.0327 (±0.0156)**  **-0.0494 (±0.0177)**  **-0.0315 (±0.0151)**  **-0.0669 (±0.0213)** | **0.0361 ***  **0.00529 ****  **0.0369 ***  **0.00165 **** |
| 1 Due to spatial autocorrelation, geographic coordinates were added as fixed variables in the full model for bird abundance | | | | |

Table S 5: Standardized estimates and standards errors (± SE) of the landscape metrics and management intensity at landscape level. Here the results of the best model (based on AICc criterion) are shown. The response variables not present in this section had a best model equal to the null model. * p < 0.05; ** p < 0.01 and *** p < 0.001**.**

| **Landscape level (N = 15)** | | | | | | |
| --- | --- | --- | --- | --- | --- | --- |
| Formula: Response variable ~ (Woodland + Crop diversity + Permanent grassland + Landscape heterogeneity) * Managt intensity | | | | | | |
|  | **Response variable** | **Explicative variables** | | **Estimate (± SE)** | | **P-value** |
| **BIRD COMMUNITY** | Shannon diversity of birds | **Landscape heterogeneity** | | **-0.0338 (±0.0094)** | | **0. 000335 ***** |
|  | Shannon diversity of insectivorous birds | **Landscape heterogeneity** | | **-0.054 (±0.0202)** | | **0.0121 **** |
| **PREDATION RATE** | Average predation | **Landscape heterogeneity** | **0.121 (±0.0433)** | | **0.00519 **** | |

Table S 6: Subset of models (ΔAICc < 2) for woodlands analyses. The response variable not present in this table (i.e. total abundance of birds) had a best model equal to the null model.

| **Formula: Shannon diversity of birds ~ (Woodland + Crop diversity + Permanent grassland) * (Managt intensity + Tree diversity)** | | | | | | | | | | | | | | | |
| --- | --- | --- | --- | --- | --- | --- | --- | --- | --- | --- | --- | --- | --- | --- | --- |
| Intercept | Managt intensity | Grassland | Woodland | Crop diversity | Tree diversity | Managt intensity:Grassland | Managt intensity:Woodland | Managt intensity:Crop diversity | Grassland:Tree diversity | Woodland:Tree diversity | Crop diversity:Tree diversity | R² | LogLik | AICc | ΔAICc |
| 2.04 | *NA* | 0.064 | *NA* | -0.061 | *NA* | *NA* | *NA* | *NA* | *NA* | *NA* | *NA* | 0.27 | 17.5 | -25.5 | 0 |
| **Formula: Shannon diversity of insectivorous birds ~ (Woodland + Crop diversity + Permanent grassland) * (Managt intensity + Tree diversity)** | | | | | | | | | | | | | | | |
| Intercept | Managt intensity | Grassland | Woodland | Crop diversity | Tree diversity | Managt intensity:Grassland | Managt intensity:Woodland | Managt intensity:Crop diversity | Grassland:Tree diversity | Woodland:Tree diversity | Crop diversity:Tree diversity | R² | LogLik | AICc | ΔAICc |
| 1.892 | *NA* | *NA* | *NA* | -0.076 | *NA* | *NA* | *NA* | *NA* | *NA* | *NA* | *NA* | 0.14 | 7.26 | -7.59 | 0.00 |
| 1.892 | -0.032 | *NA* | *NA* | -0.083 | *NA* | *NA* | *NA* | *NA* | *NA* | *NA* | *NA* | 0.15 | 7.67 | -5.73 | 1.86 |
| 1.892 | *NA* | *NA* | *NA* | *NA* | *NA* | *NA* | *NA* | *NA* | *NA* | *NA* | *NA* | 0 | 5.08 | -5.72 | 1.88 |
| **Formula: Caterpillar predation ~ (Woodland + Crop diversity + Permanent grassland) * (Managt intensity + Tree diversity)** | | | | | | | | | | | | | | | |
| Intercept | Managt intensity | Grassland | Woodland | Crop diversity | Tree diversity | Managt intensity:Grassland | Managt intensity:Woodland | Managt intensity:Crop diversity | Grassland:Tree diversity | Woodland:Tree diversity | Crop diversity:Tree diversity | R² | LogLik | AICc | ΔAICc |
| 0.128 | 0.030 | -0.038 | 0.066 | *NA* | *NA* | *NA* | 0.098 | *NA* | *NA* | *NA* | *NA* | 0.36 | 29.09 | -42.5 | 0.00 |
| 0.127 | *NA* | *NA* | 0.039 | *NA* | -0.042 | *NA* | *NA* | *NA* | *NA* | *NA* | *NA* | 0.19 | 25.70 | -41.8 | 0.74 |
| 0.127 | 0.035 | -0.047 | *NA* | 0.037 | *NA* | *NA* | *NA* | *NA* | *NA* | *NA* | *NA* | 0.27 | 27.12 | -41.7 | 0.79 |
| 0.128 | 0.028 | *NA* | 0.071 | *NA* | -0.035 | *NA* | 0.087 | *NA* | *NA* | *NA* | *NA* | 0.34 | 28.67 | -41.7 | 0.85 |
| 0.127 | *NA* | -0.041 | *NA* | *NA* | *NA* | *NA* | *NA* | *NA* | *NA* | *NA* | *NA* | 0.12 | 24.30 | -41.7 | 0.86 |
| 0.127 | *NA* | -0.031 | 0.035 | *NA* | -0.035 | *NA* | *NA* | *NA* | *NA* | *NA* | *NA* | 0.27 | 26.98 | -41.5 | 1.07 |
| 0.127 | *NA* | -0.045 | *NA* | 0.030 | *NA* | *NA* | *NA* | *NA* | *NA* | *NA* | *NA* | 0.19 | 25.46 | -41.3 | 1.22 |
| 0.128 | 0.029 | *NA* | 0.070 | *NA* | *NA* | *NA* | 0.101 | *NA* | *NA* | *NA* | *NA* | 0.26 | 26.85 | -41.2 | 1.33 |
| 0.127 | *NA* | -0.038 | 0.028 | *NA* | *NA* | *NA* | *NA* | *NA* | *NA* | *NA* | *NA* | 0.18 | 25.33 | -41.1 | 1.47 |
| 0.127 | *NA* | -0.035 | *NA* | *NA* | -0.029 | *NA* | *NA* | *NA* | *NA* | *NA* | *NA* | 0.18 | 25.32 | -41.0 | 1.50 |
| 0.127 | 0.034 | -0.042 | *NA* | 0.038 | -0.028 | *NA* | *NA* | *NA* | *NA* | *NA* | *NA* | 0.33 | 28.32 | -41.0 | 1.56 |
| 0.127 | 0.027 | -0.042 | *NA* | *NA* | *NA* | *NA* | *NA* | *NA* | *NA* | *NA* | *NA* | 0.18 | 25.26 | -40.9 | 1.61 |
| 0.132 | *NA* | *NA* | 0.046 | *NA* | -0.038 | *NA* | *NA* | *NA* | *NA* | -0.029 | *NA* | 0.25 | 26.68 | -40.9 | 1.68 |
| 0.127 | *NA* | -0.040 | *NA* | 0.031 | -0.029 | *NA* | *NA* | *NA* | *NA* | *NA* | *NA* | 0.25 | 26.62 | -40.7 | 1.79 |
| 0.127 | *NA* | -0.035 | 0.033 | 0.028 | -0.036 | *NA* | *NA* | *NA* | *NA* | *NA* | *NA* | 0.32 | 28.19 | -40.7 | 1.81 |
| 0.127 | *NA* | *NA* | *NA* | *NA* | -0.035 | *NA* | *NA* | *NA* | *NA* | *NA* | *NA* | 0.091 | 23.79 | -40.7 | 1.88 |
| 0.132 | *NA* | -0.032 | 0.042 | NA | -0.031 | *NA* | *NA* | *NA* | *NA* | -0.030 | *NA* | 0.32 | 28.14 | -40.6 | 1.90 |
| 0.127 | 0.025 | *NA* | 0.039 | NA | -0.041 | *NA* | *NA* | *NA* | *NA* | *NA* | *NA* | 0.24 | 26.56 | -40.6 | 1.91 |
| 0.120 | *NA* | -0.036 | NA | 0.036 | -0.020 | *NA* | *NA* | *NA* | 0.038 | *NA* | *NA* | 0.32 | 28.12 | -40.6 | 1.94 |
| 0.127 | 0.035 | -0.045 | 0.025 | 0.035 | *NA* | *NA* | *NA* | *NA* | *NA* | *NA* | *NA* | 0.32 | 28.12 | -40.6 | 1.95 |

Table S 7: Subset of models (ΔAICc < 2) for cereal fields analyses. The response variables not present in this table (i.e. egg and seed predation) had a best model equal to the null model.

| **Formula: Shannon diversity of birds ~ (Woodland + Crop diversity + Permanent grassland) * (Managt intensity + Plant diversity)** | | | | | | | | | | | | | | | | | | | | | | | | | | | | | | | | |
| --- | --- | --- | --- | --- | --- | --- | --- | --- | --- | --- | --- | --- | --- | --- | --- | --- | --- | --- | --- | --- | --- | --- | --- | --- | --- | --- | --- | --- | --- | --- | --- | --- |
| Intercept | Plant diversity | | Grassland | | Woodland | | Crop diversity | | | Managt intensity | | | Plant diversity:Grassland | | | | Plant diversity:Woodland | | | Plant diversity:Crop diversity | | Grassland:Managt intensity | | Woodland: Managt intensity | | Crop diversity: Managt intensity | | R² | LogLik | AICc | ΔAICc | |
| 2.22 | *NA* | | 0.089 | | *NA* | | *NA* | | | *NA* | | | *NA* | | | | *NA* | | | *NA* | | *NA* | | *NA* | | *NA* | | 0.17 | 6.19 | -5.42 | 0.00 | |
| 2.22 | *NA* | | 0.112 | | *NA* | | *NA* | | | 0.062 | | | *NA* | | | | *NA* | | | *NA* | | *NA* | | *NA* | | *NA* | | 0.23 | 7.45 | -5.23 | 0.19 | |
| 2.20 | *NA* | | 0.102 | | *NA* | | *NA* | | | 0.051 | | | *NA* | | | | *NA* | | | *NA* | | -0.049 | | *NA* | | *NA* | | 0.27 | 8.24 | -3.87 | 1.55 | |
| **Formula: Shannon diversity of insectivorous birds ~ (Woodland + Crop diversity + Permanent grassland) * (Managt intensity + Plant diversity)** | | | | | | | | | | | | | | | | | | | | | | | | | | | | | | | | |
| Intercept | Plant diversity | | Grassland | | Woodland | | Crop diversity | | | Managt intensity | | | | Plant diversity:Grassland | | | Plant diversity:Woodland | | | Plant diversity:Crop diversity | | Grassland:Managt intensity | | Woodland: Managt intensity | | Crop diversity: Managt intensity | | R² | LogLik | AICc | ΔAICc | |
| 1.48 | -0.041 | | 0.073 | | *NA* | | *NA* | | | 0.108 | | | | 0.098 | | | *NA* | | | *NA* | | *NA* | | *NA* | | *NA* | | 0.43 | 10.6 | -5.29 | 0.00 | |
| 1.48 | *NA* | | 0.103 | | *NA* | | *NA* | | | 0.118 | | | | *NA* | | | *NA* | | | *NA* | | *NA* | | *NA* | | *NA* | | 0.29 | 7.39 | -5.10 | 0.19 | |
| **Formula: Total abundance of birds ~ (Woodland + Crop diversity + Permanent grassland) * (Managt intensity + Plant diversity)** | | | | | | | | | | | | | | | | | | | | | | | | | | | | | | | | |
| Intercept | Plant diversity | Grassland | | Woodland | | Crop diversity | | | Managt intensity | | | Xl93m | | | | Yl93m | | Plant diversity:Grassland | Plant diversity:Woodland | | Plant diversity:Crop diversity | | Grassland:Managt intensity | | Woodland:Managt intensity | | Crop diversity:Managt intensity | R² | LogLik | AICc | | ΔAICc |
| 2.58 | *NA* | 0.098 | | *NA* | | *NA* | | | *NA* | | | *NA* | | | | -0.153 | | *NA* | *NA* | | *NA* | | *NA* | | *NA* | | *NA* | 0.39 | -71.4 | 149.8 | | 0.00 |
| 2.58 | *NA* | *NA* | | *NA* | | *NA* | | | *NA* | | | *NA* | | | | -0.177 | | *NA* | *NA* | | *NA* | | *NA* | | *NA* | | *NA* | 0.32 | -73.0 | 150.5 | | 0.67 |
| 2.58 | *NA* | 0.102 | | *NA* | | *NA* | | | *NA* | | | -0.070 | | | | -0.125 | | *NA* | *NA* | | *NA* | | *NA* | | *NA* | | *NA* | 0.42 | -70.6 | 151.0 | | 1.15 |
| 2.58 | *NA* | *NA* | | *NA* | | *NA* | | | *NA* | | | -0.062 | | | | -0.152 | | *NA* | *NA* | | *NA* | | *NA* | | *NA* | | *NA* | 0.35 | -72.4 | 151.7 | | 1.92 |
| **Formula: Shannon diversity of carabids ~ (Woodland + Crop diversity + Permanent grassland) * (Managt intensity + Plant diversity)** | | | | | | | | | | | | | | | | | | | | | | | | | | | | | | | | |
| Intercept | Plant diversity | | Grassland | | Woodland | | | Crop diversity | | | Managt intensity | | | | Plant diversity:Grassland | | Plant diversity:Woodland | | | Plant diversity:Crop diversity | | Grassland:Managt intensity | | Woodland: Managt intensity | | Crop diversity: Managt intensity | | R² | LogLik | AICc | ΔAICc | |
| 1.25 | *NA* | | *NA* | | *NA* | | | *NA* | | | -0.20 | | | | *NA* | | *NA* | | | *NA* | | *NA* | | *NA* | | *NA* | | 0.17 | -16.7 | 40.3 | 0.00 | |
| 1.25 | *NA* | | *NA* | | 0.07 | | | *NA* | | | -0.19 | | | | *NA* | | *NA* | | | *NA* | | *NA* | | *NA* | | *NA* | | 0.19 | -16.3 | 42.2 | 1.85 | |
| 1.25 | *NA* | | -0.07 | | *NA* | | | *NA* | | | -0.22 | | | | *NA* | | *NA* | | | *NA* | | *NA* | | *NA* | | *NA* | | 0.19 | -16.3 | 42.3 | 1.94 | |
| **Formula: Abundance of carabids ~ (Woodland + Crop diversity + Permanent grassland) * (Managt intensity + Plant diversity)** | | | | | | | | | | | | | | | | | | | | | | | | | | | | | | | | |
| Intercept | Plant diversity | | Grassland | | Woodland | | | Crop diversity | | | Managt intensity | | | | Plant diversity:Grassland | | Plant diversity:Woodland | | | Plant diversity:Crop diversity | | Grassland:Managt intensity | | Woodland: Managt intensity | | Crop diversity: Managt intensity | | R² | LogLik | AICc | ΔAICc | |
| 3.96 | 0.40 | | 0.35 | | 0.44 | | *NA* | | | *NA* | | | *NA* | | | | *NA* | | | *NA* | | *NA* | | *NA* | | 0.44 | | 0.44 | -143.1 | 298.7 | 0.00 | |
| **Formula: Aphid predation (crop-level)~ (Woodland + Crop diversity + Permanent grassland) * (Managt intensity + Plant diversity)** | | | | | | | | | | | | | | | | | | | | | | | | | | | | | | | | |
| Intercept | Plant diversity | | Grassland | | Woodland | | Crop diversity | | | Managt intensity | | | Plant diversity:Grassland | | | | Plant diversity:Woodland | | | Plant diversity:Crop diversity | | Grassland:Managt intensity | | Woodland: Managt intensity | | Crop diversity: Managt intensity | | R² | LogLik | AICc | ΔAICc | |
| 0.156 | *NA* | | *NA* | | -0.035 | | *NA* | | | *NA* | | | *NA* | | | | *NA* | | | *NA* | | *NA* | | *NA* | | *NA* | | 0.16 | 33.4 | -59.8 | 0.00 | |
| 0.156 | *NA* | | *NA* | | -0.041 | | -0.020 | | | *NA* | | | *NA* | | | | *NA* | | | *NA* | | *NA* | | *NA* | | *NA* | | 0.21 | 34.2 | -58.8 | 1.01 | |
| 0.156 | *NA* | | -0.014 | | -0.034 | | *NA* | | | *NA* | | | *NA* | | | | *NA* | | | *NA* | | *NA* | | *NA* | | *NA* | | 0.19 | 33.8 | -58.1 | 1.72 | |
| 0.156 | 0.014 | | *NA* | | -0.039 | | *NA* | | | *NA* | | | *NA* | | | | *NA* | | | *NA* | | *NA* | | *NA* | | *NA* | | 0.18 | 33.8 | -58.0 | 1.85 | |
| 0.156 | *NA* | | *NA* | | -0.036 | | *NA* | | | -0.013 | | | *NA* | | | | *NA* | | | *NA* | | *NA* | | *NA* | | *NA* | | 0.18 | 33.7 | -57.9 | 1.92 | |
| **Formula: Aphid predation (ground-level)~ (Woodland + Crop diversity + Permanent grassland) * (Managt intensity + Plant diversity)** | | | | | | | | | | | | | | | | | | | | | | | | | | | | | | | | |
| Intercept | Plant diversity | | Grassland | | Woodland | | Crop diversity | | | Managt intensity | | | Plant diversity:Grassland | | | | Plant diversity:Woodland | | | Plant diversity:Crop diversity | | Grassland:Managt intensity | | Woodland: Managt intensity | | Crop diversity: Managt intensity | | R² | LogLik | AICc | ΔAICc | |
| 0.910 | -0.033 | | *NA* | | *NA* | | -0.049 | | | -0.031 | | | *NA* | | | | *NA* | | | *NA* | | *NA* | | *NA* | | -0.067 | | 0.38 | 33.3 | -50.9 | 0.00 | |
| 0.910 | *NA* | | *NA* | | *NA* | | -0.040 | | | -0.026 | | | *NA* | | | | *NA* | | | *NA* | | *NA* | | *NA* | | -0.067 | | 0.29 | 31.2 | -49.9 | 0.95 | |
| 0.910 | *NA* | | *NA* | | -0.028 | | -0.052 | | | -0.028 | | | *NA* | | | | *NA* | | | *NA* | | *NA* | | *NA* | | -0.076 | | 0.35 | 32.6 | -49.5 | 1.35 | |

Table S 8: Subsets of models (ΔAICc < 2) for matched landscapes analyses. The response variable not present in this table (i.e. total abundance of birds) had a best model equal to the null model.

| **Formula: Shannon diversity of birds~ (Woodland + Crop diversity + Permanent grassland + Landscape heterogeneity) * Managt intensity** | | | | | | | | | | | | | | |
| --- | --- | --- | --- | --- | --- | --- | --- | --- | --- | --- | --- | --- | --- | --- |
| Intercept | Managt intensity | Grassland | Woodland | Crop diversity | Landscape heterogeneity | Managt intensity:Grassland | Managt intensity:Woodland | Managt intensity:Crop diversity | Managt intensity: Landscape heterogeneity | R² | LogLik | AICc | ΔAICc |  |
| 0*.*89 | *NA* | *NA* | *NA* | *NA* | -0*.*03 | *NA* | *NA* | *NA* | *NA* | 0.46 | 28*.*9 | -49*.*6 | 0*.*00 |  |
| **Formula: Shannon diversity of insectivorous birds~ (Woodland + Crop diversity + Permanent grassland + Landscape heterogeneity) * Managt intensity** | | | | | | | | | | | | | | |
| Intercept | Managt intensity | Grassland | Woodland | Crop diversity | Landscape heterogeneity | Managt intensity:Grassland | Managt intensity:Woodland | Managt intensity:Crop diversity | Managt intensity: Landscape heterogeneity | R² | LogLik | AICc | ΔAICc |  |
| 0*.*63 | *NA* | *NA* | *NA* | *NA* | -0*.*054 | *NA* | *NA* | *NA* | *NA* | 0.32 | 17*.*4 | -26*.*6 | 0*.*0 |  |
| **Formula: Average predation rate ~ (Woodland + Crop diversity + Permanent grassland + Landscape heterogeneity) * Managt intensity** | | | | | | | | | | | | | | |
| Intercept | Managt intensity | Grassland | Woodland | Crop diversity | Landscape heterogeneity | Managt intensity:Grassland | Managt intensity:Woodland | Managt intensity:Crop diversity | Managt intensity: Landscape heterogeneity | R² | LogLik | AICc | ΔAICc |  |
| 0*.*47 | *NA* | *NA* | *NA* | *NA* | 0*.*12 | *NA* | *NA* | *NA* | *NA* | 0.34 | 6*.*01 | -3*.*85 | 0*.*00 |  |
| 0*.*47 | *NA* | *NA* | 0*.*06 | *NA* | 0*.*11 | *NA* | *NA* | *NA* | *NA* | 0.42 | 6*.*98 | -1*.*95 | 1*.*89 |  |

1. Larrieu L, Gonin P. L’indice de biodiversité potentielle (ibp) : une méthode simple et rapide pour évaluer la biodiversité potentielle des peuplements forestiers. Rev For Fr [Internet]. 2008;(6). Available from: https://hal.archives-ouvertes.fr/hal-03449570

2. Larrieu L, Cabanettes A, Gouix N, Burnel L, Bouget C, Deconchat M. Post-harvesting dynamics of the deadwood profile: the case of lowland beech-oak coppice-with-standards set-aside stands in France. Eur J Forest Res. 2019 Apr 1;138(2):239–51.

3. Larrieu L, Cabanettes A, Gouix N, Burnel L, Bouget C, Deconchat M. Development over time of the tree-related microhabitat profile: the case of lowland beech–oak coppice-with-standards set-aside stands in France. Eur J Forest Res. 2017 Feb 1;136(1):37–49.

4. McHugh NM, Moreby S, Lof ME, Werf W, Holland JM. The contribution of semi‐natural habitats to biological control is dependent on sentinel prey type. Corley J, editor. J Appl Ecol. 2020 May;57(5):914–25.

5. Ricci B, Lavigne C, Alignier A, Aviron S, Biju-Duval L, Bouvier JC, et al. Local pesticide use intensity conditions landscape effects on biological pest control. Proc R Soc B. 2019 Jun 12;286(1904):20182898.

6. Baselga A, Orme CDL. betapart: an R package for the study of beta diversity. Methods in Ecology and Evolution. 2012;3(5):808–12.

7. Baselga A. Partitioning abundance-based multiple-site dissimilarity into components: balanced variation in abundance and abundance gradients. Methods in Ecology and Evolution. 2017;8(7):799–808.
